# Study protocol: a comprehensive multi-method neuroimaging approach to disentangle developmental effects and individual differences in second language learning

**DOI:** 10.1186/s40359-022-00873-x

**Published:** 2022-07-08

**Authors:** W. M. Menks, C. Ekerdt, G. Janzen, E. Kidd, K. Lemhöfer, G. Fernández, J. M. McQueen

**Affiliations:** 1 Donders Institute for Brain, Cognition and Behaviour, Radboud University, and Radboud University Medical Centre, Nijmegen, the Netherlands; 2 Max Planck Institute for Psycholinguistics, Nijmegen, the Netherlands; 3 Behavioural Science Institute, Radboud University, Nijmegen, the Netherlands; 4 ARC Centre of Excellence for the Dynamics of Language, Canberra, Australia; 5 Research School of Psychology, Australian National University, Canberra, Australia

**Keywords:** Second language acquisition, Grammar learning, Word learning, Individual differences, Development, Functional magnetic resonance imaging (fMRI), White matter microstructure, Diffusion weighted magnetic resonance imaging, Resting-state connectivity, Structural neuroimaging

## Abstract

**Background:**

While it is well established that second language (L2) learning success changes with age and across individuals, the underlying neural mechanisms responsible for this developmental shift and these individual differences are largely unknown. We will study the behavioral and neural factors that subserve new grammar and word learning in a large cross-sectional developmental sample. This study falls under the *NWO* (Nederlandse Organisatie voor Wetenschappelijk Onderzoek [Dutch Research Council]) Language in Interaction consortium (website: https://www.languageininteraction.nl/).

**Methods:**

We will sample 360 healthy individuals across a broad age range between 8 and 25 years. In this paper, we describe the study design and protocol, which involves multiple study visits covering a comprehensive behavioral battery and extensive magnetic resonance imaging (MRI) protocols. On the basis of these measures, we will create behavioral and neural fingerprints that capture age-based and individual variability in new language learning. The behavioral fingerprint will be based on first and second language proficiency, memory systems, and executive functioning. We will map the neural fingerprint for each participant using the following MRI modalities: T1‐weighted, diffusion-weighted, resting-state functional MRI, and multiple functional-MRI paradigms. With respect to the functional MRI measures, half of the sample will learn grammatical features and half will learn words of a new language. Combining all individual fingerprints allows us to explore the neural maturation effects on grammar and word learning.

**Discussion:**

This will be one of the largest neuroimaging studies to date that investigates the developmental shift in L2 learning covering preadolescence to adulthood. Our comprehensive approach of combining behavioral and neuroimaging data will contribute to the understanding of the mechanisms influencing this developmental shift and individual differences in new language learning. We aim to answer: (I) do these fingerprints differ according to age and can these explain the age-related differences observed in new language learning? And (II) which aspects of the behavioral and neural fingerprints explain individual differences (across and within ages) in grammar and word learning? The results of this study provide a unique opportunity to understand how the development of brain structure and function influence new language learning success.

**Supplementary Information:**

The online version contains supplementary material available at 10.1186/s40359-022-00873-x.

## Background

One estimate suggests that more than half of the world’s population is using a second language in addition to their native tongue [1]. Foreign languages are thought to be very useful for self-development and employment; recently, the European Union has set the objective that every young European should learn two foreign European languages [2]. For decades, behavioral studies have observed that learning a second language (L2) is more successful when starting at a young age. This presents a paradox, since adults’ general cognitive and language abilities are superior to children, leading one to expect that adults would be better at language learning than children. Subsequently, the hypothesis of a biological sensitive period for L2 learning emerged: native-like L2 proficiency is feasible when learned within a specific age range [3–10]. L2 learning ability generally declines between late childhood and late adolescence [4, 6, 7, 11]. Although children are slower in the initial stage of L2 learning compared to adults, children more often reach native-like proficiency in their L2, which is seldom observed for adult learners [5, 7, 12, 13]. For grammar learning, a recent large-scale study observed a strong decline in L2 attainment starting from the age of 17 years [11]. This supports the existence of a sensitive period for L2 grammar learning, although the predicted age of offset is much later than previously proposed. Behaviorally, these differences in how children and adults learn an L2 have been investigated, but the underlying developmental mechanisms are not thoroughly understood.

In addition to age-related variability, most studies have also observed individual differences in L2 attainment, with evidence that some late learners can obtain near-native-like proficiency [12]. Therefore, researchers have suggested that other factors—besides age—may underlie L2 learning success [14–16]. L2 learning consists of multiple interconnected complex language domains (e.g., grammar, semantics, phonology) that are supported by general-purpose cognitive functions such as working memory and long-term declarative and procedural memory processes. The complex interplay between individual differences in language and cognitive abilities and developmental factors pose a challenge for researchers in the field of L2 learning variability. In this protocol, we will present a study that focuses on two major language domains—grammar and words—in relation to age and individual variability in L2 learning.

### The cognitive fingerprint of L2 learning variability

#### Age-related variability in L2 learning

Behavioral studies have extensively investigated age-related variability in L2 learning in the last decades, and pointed towards maturational constraints as its underlying cause. Whether the observed L2 variability is caused by maturational constraints affecting specialized language processes or more general cognitive processes is still under debate [7, 10, 13, 17]. Thus far, studies have found age-related variations in general cognitive processes that correlate with L2 proficiency, such as long-term memory, short-term memory capacity, and cognitive control [18–21]. Interestingly, in most of these cognitive domains, children are much less capable than adults, as competence in these domains increases with age [22, 23]. Researchers have proposed several theories that link L2 learning with declarative and procedural memory abilities [24–26]. According to these theories, different memory systems are involved depending on the language domain: for example, word learning in general involves declarative (i.e., explicit) memory ability, whereas grammar learning relies mostly on procedural (i.e., implicit) memory ability. In line with this model, several behavioral studies in children have reported correlations between first language (L1) vocabulary proficiency and declarative learning abilities, and linked L1 grammar learning success with procedural memory abilities [27, 28]. For L2 grammar learning, however, it has been proposed that children and adults might use different memory processes [29]. Children use their early-maturated procedural memory, while adults are thought to rely more on their declarative memory abilities [5, 30–33]. The model aligns with the developmental trajectories of the two memory systems: procedural learning abilities mature much earlier—prepubertally—in comparison to declarative learning abilities and working memory that both have a prolonged maturation trajectory into young adulthood [34–37]. In L2 word learning, both children and adults rely on the declarative memory system. However, some factors that influence word learning do so differently during different times in development. Two factors that influence declarative memory and which have shown developmental differences are prior knowledge and memory consolidation. While adults, in general, have larger vocabularies and therefore more prior knowledge to benefit from during word learning, children profit more from memory consolidation during word learning [29, 31, 38–41]. In sum, maturational changes could affect which memory system is utilized and how prior knowledge and consolidation benefit L2 learning; however, the underlying mechanisms have not been thoroughly investigated in relation to age-related variability in L2 learning.

In addition to maturational constraints, environmental factors could partially explain the observed age-related L2 learning variability. In particular, the manner of L2 learning, L2 input quality, and amount of L2 exposure could influence how well a new language is learned [5, 42]. The manner of L2 learning could influence which memory systems are utilized. L2 learning through explicit classroom instruction, for instance, will depend more on the declarative memory system, while immersion-like L2 input will rely more on the procedural memory system [43, 44]. Adults often learn a new L2 through classroom instruction, which could make them worse L2 learners compared to early L2 learners who often acquired their L2 through immersion. Furthermore, quantitative and qualitative differences in L2 input are thought to exist between young and adult L2 learners. Children, for example, might receive not only more but also more simplified L2 input, which could benefit L2 learning; adults on the other hand typically receive more complex L2 input that could interfere with their L2 acquisition. To what extent these environmental factors influence L2 learning, independent of the maturational constraints, is not yet fully understood.

In sum, researchers have suggested several maturational constraints and environmental factors that could underlie the age-related variability in L2 learning. Until now, L2 studies have often solely focused on one or two factors such as L2 age of acquisition (AoA) and/or L2 exposure, leaving out other latent factors. As a consequence, there is little consensus which factors are the best predictors for L2 learning success during development. To find an answer to the underlying mechanisms of age-related L2 learning variability, an interdisciplinary approach, across psycholinguistics, memory, and (neuro)development, is essential to investigate all the aforementioned factors.

#### Individual variability in L2 learning

Apart from age-related variability, interindividual variability exists between L2 learners [14, 45]. This individual L2 learning success could be influenced by a multitude of external and internal factors: language aptitude, motivation, L2 exposure, learning strategies, L1 proficiency, education, and socioeconomic factors [45–50]. Especially domain-general factors within language aptitude—i.e., cognitive control, intelligence, and memory—are thought to be the most substantial predictors of L2 learning success [14, 47, 51, 52]. In particular, domain-general memory systems are thought to be highly related to L2 learning success; for example, a positive effect of (verbal and nonverbal) working memory was found for adult L2 learning outcomes [53–56]. Likewise, L2 learning success in adults is frequently linked with greater episodic and procedural memory abilities [30, 57–59]. Furthermore, inhibitory control ability, an executive functioning skill, is another domain-general factor that can predict L2 learning success in adults [60]. Environmental or external factors, for example, socioeconomic status, L2 exposure, and education, shape early language learning experiences and are particularly relevant for L2 learning in young individuals [61, 62]. In sum, past research has identified many external and internal factors that could underlie L2 learning success. To date, the aforementioned factors have not often been simultaneously examined, and thus it remains unknown which factors are most predictive for L2 learning success. Furthermore, L2 studies frequently investigated individual differences within monolingual and bilingual groups without taking age, or age of onset (AoA), into consideration. Many domain-general cognitive processes, that may underlie these individual differences, are still under development during adolescence; therefore, age should be taken into account when investigating these individual differences.

### The neural fingerprint of L2 learning variability

#### The neural correlates underlying L2 learning

In the past decades, the usage of various magnetic resonance imaging (MRI) methods have enabled researchers to shed light on the neural correlates underlying L2 word and grammar learning [48, 63–65]. For instance, functional MRI (fMRI) studies have repeatedly observed increased brain activation during L2 word and grammar learning tasks: especially within frontal (i.e., inferior frontal gyrus (IFG)) and parietal brain regions, as well as within subcortical structures like the basal ganglia and cerebellum [48, 65]. Moreover, each L2 subdomain additionally recruits distinctive brain regions during L2 learning: for example, the middle temporal gyrus (MTG) and superior temporal gyrus (STG) and the hippocampus are commonly activated during L2 word learning [66–68], whereas the basal ganglia are often recruited during L2 grammar learning [65, 69, 70].

Besides task-based fMRI, L2 studies have used functional connectivity analyses by means of resting-state fMRI (rsfMRI), to understand how the brain areas implicated in L2 learning interact and are co-activated in larger functional brain networks [71–74]. Most rsfMRI studies have specially focused on the connections between the bilateral IFG and other language-related brain regions [75]. Additionally, interhemispheric coupling of the bilateral IFG is associated with both L2 performance [71] and AoA [72, 76]. These findings are in line with fMRI studies that linked brain activation patterns within the IFG and other language-related regions, e.g., the middle frontal gyrus (MFG) and STG, with L2 proficiency and/or AoA [64, 77, 78].

Besides altered brain activation, L2 learning is additionally associated with structural changes within specific gray and white matter regions of the brain. Increased gray matter density and cortical thickness is, for example, observed within several cortical regions (i.e., IFG, MTG, STG, MFG, and inferior parietal lobe (IPL)) in relation to L2 learning [79–83]. Within these cortical regions, both gray matter density and thickness have been linked to L2 proficiency and AoA [48, 79, 80, 83]. Moreover, underneath most of these cortical brain regions, white matter alterations have been observed that were linked to L2 learning [64, 79, 84]. For example, L2 learning ability is related to microstructural alterations within the arcuate fasciculus (AF), an important white matter tract that connects the temporal and inferior parietal cortex to the frontal lobe [85–87]. Other white matter tracts that are frequently linked to L2 learning success are the inferior fronto-occipital fasciculus (IFOF) and the superior longitudinal fasciculus (SLF) [84, 88–90]. Taken together, a multitude of neuroimaging studies have proposed several neural components that each could predict L2 learning success. No study to date, however, has examined the aforementioned neural components within one large homogenous sample. It therefore remains unanswered to which extent each neural component predicts L2 learning success, and how it contributes to individual variability in L2 learning.

#### The neural correlates of age-related variability in L2 learning

A number of adult and developmental neuroimaging studies provide insight into the neural bases of age-related variability in L2, but a comprehensive overview of how these neural correlates change during development is lacking. In the past, studies have used AoA within adult samples to indirectly investigate the effect of neural maturation on L2 learning success [83, 91–93]. AoA remains a relatively imprecise predictor for age-related variability, especially considering that it correlates with other L2 factors that influence L2 learning such as L2 input, L2 exposure, and L2 usage [5, 42, 70]. Therefore, ideally, neuroimaging studies should rather focus on age directly, as a gradient, within a large—linguistically homogeneous—sample [94]. Several developmental studies have looked at the neural correlates of L2 learning. Their results demonstrated activation within the IFG, STG, and MTG and less frequently within the insula, cingulate cortex, basal ganglia and cerebellum during L2 processing [95–97]. Although these brain activation patterns overlap to some extent with the L2 language-related regions identified in adults, age differences still appear to exist [65]. For example, two studies using between group analyses revealed different L2 brain activation patterns for adults and late adolescents in comparison to younger teenagers [96, 97]. Thus far, one developmental study has implemented correlational analyses within a developmental sample covering 8 to 18 years to investigate age effects on L2 processing of phonology [98]. The hemodynamic response during L2 phonological processing showed a positive linear relationship with age within the IFG. Together, these findings support the idea that developmental changes in neural correlates underlying L2 processes, and thus L2 learning, exist and potentially can explain differences in L2 learning success. To achieve a more comprehensive understanding of the developmental trajectory of the neural correlates underlying L2 learning success, neuroimaging studies should investigate children and adults within a large sample that has an age range that covers the hypothesized sensitive period for L2 learning. Only then can age-related changes in functional activation be investigated in more detail and compared to L2 learning success.

#### Brain maturation and age-related variability in L2 learning

To disentangle the neural mechanisms underlying age-related variability in L2 learning, it is important to understand the overall maturation trajectory of the brain. In general, gray matter volume increases during early childhood, after which it gradually decreases during puberty and early adulthood [99, 100]. This decrease is thought to be a correlate of pruning of excess neurons and is therefore often used as a direct proxy of brain maturation [100, 101]. The starting point and rate of gray matter decrease is not uniform, but varies between different regions of the brain. In particular, the prefrontal cortex and temporal lobes follow the most prolonged maturation trajectory, one that ends around 20 years of age [102–104]. These late-maturing brain regions are linked with language and memory processes. Although this has not been thoroughly investigated, several studies have associated gray matter maturation with age-related variability in working memory and cognitive control [22, 105–108], but also language-specific processes [109, 110]. In contrast to gray matter, white matter volume increases during adolescence, reflecting increased myelination and axon thickness, while the overall brain volume remains stable [111–114]. The white matter tracts that are commonly linked to L1 language processes (i.e., AF, SLF, IFOF) have a protracted maturation trajectory that peaks around early adulthood [115–119]. In addition to these structural changes, brain maturation leads to changes in functional activation. For example, brain activation patterns seem to shift from a diffuse to a more focal pattern in relation to age [120–123]. This shift is seen as the increasing functional specialization and reorganization of the brain with age. The prolonged and gradual maturation of prefrontal and temporal regions may therefore contribute to age-related variability in L2 learning success, since domain-general and language-specific L2 learning processes are seated in these regions [124, 125]. Although knowledge about the prolonged brain maturation in general is increasing, still little is known about how the developmental trajectory of domain-general and language-specific processes relates to L2 learning ability.

## Current study

There is a paradox in L2 learning research: Adults’ general cognitive and language abilities are superior to children’s, but adults are nonetheless outperformed by children in L2 acquisition. It is well established that children and adults differ in their language learning abilities; but how it is reflected in the neural mechanisms underlying new language learning^1^ is poorly understood. To date, studies that have explored the neural correlates of L2 learning have typically used small samples and have made group comparisons across wide age ranges. This limited the possibilities to explore potential changes in L2 learning between childhood and early adulthood. Additionally, most neuroimaging studies researching L2 learning have focused on a single MRI technique to investigate either brain activation, through task-based or resting-state fMRI, or brain structure such as gray matter structure or white matter connectivity. We will combine all these neuroimaging techniques to comprehensively investigate the neural correlates underlying new language learning in a cross-sectional sample. The key strength of our study is our focus on age-related as well as individual differences: using an extensive behavioral testing battery, we will create both a behavioral and a neural fingerprint of every individual to determine how each subcomponent affects L2 learning at different ages.

Our study is the first large-scale neurodevelopmental study investigating the neural correlates underlying aspects of new language learning across a broad developmental age-range. We are collecting a dataset including structural and functional MR modalities, as well as an in-depth cognitive profile. With this comprehensive dataset, we can answer questions about the development of the neural correlates underlying L2 learning that have not been explored thus far. Our objective is to explain the widely reported age-related behavioral differences in L2 learning success. Additionally, we aim to examine whether the previously reported sensitive period for grammar learning can also be observed neurally. Our state-of-the art dataset will enable us to test a large number of hypotheses. One of our main questions is to examine how brain activation patterns in response to new language learning will differ in adults and children. We hypothesize that children will recruit a larger network of brain regions as compared to adults [126]. The second aim of our study is to explore individual differences in new language learning success. Here, we will investigate which combination of neural and behavioral factors serves as the best predictor for this individual variability, independent of age. With regard to this objective, we hypothesize that new language learning success is positively linked to L1 language aptitude and memory skills, as well as the white matter connectivity within the language-network.

## Methods/design

### Participants

#### Sample size and recruitment

This project plans to recruit 360 Dutch native speakers from the ages 8 to 25 years from the general population. We aim to distribute all 360 participants evenly over this age range to have enough power to detect both age-related variability and individual variability. Adults will be partly recruited through a specialized research participation system at the Radboud University, and we will additionally promote our study through posters at university faculties, supermarkets, and social media posts. Young participants and their caregivers will be reached through flyers and posters in their schools, libraries, sport clubs, and community events. Individuals choose to take part in this study voluntarily, so that we will create a self-selecting sample. Data collection has started in 2019 and is projected to end in 2023.

#### Inclusion and exclusion criteria

To obtain a homogenous sample in relation to essential language factors (i.e., native tongue, bilingualism, and exposure to foreign languages) several strict inclusion criteria have been chosen for this study. First, only right-handed Dutch native speakers who were raised monolingually up to the age of 6 are eligible for inclusion. Generally, Dutch children formally start to learn English around the age of 8 and are regularly exposed to English through television, internet, and radio, whereas other foreign languages are generally learned at high school when children are around the age of 12. For this reason, we expect that all participants will have learned or will have been exposed to English when taking part in our study, and thus we will consider English as their second language. To study new language learning, we will train participants on two unfamiliar natural languages, namely Finnish and Icelandic. We will only include individuals who are unfamiliar with these two natural languages. Additionally, only individuals who have no history of neurological or psychiatric treatment/illness, a current psychiatric diagnosis, language impairments or learning disabilities will be included. Importantly, only individuals that have no MR contraindications can participate in this study. All adult participants have to give written informed consent; for the young participants, the caregivers have to give written informed consent.

### Procedure

#### Flowchart for subprojects

**Fig. 1:**
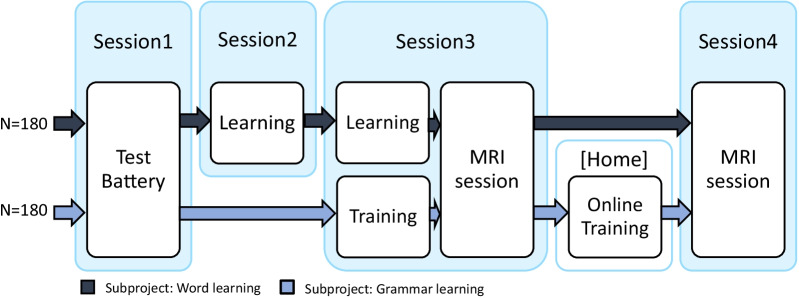
Study flowchart for each subproject: word project (dark blue arrow), grammar (light blue arrow). Each subproject will include a sample of 180 participants between the ages 8 and 25 years. All 4 sessions, except the home training, will take place at the Donders Institute. In the word project (dark blue arrows), following the test battery during session 1, participants will return to the Donders Institute to learn the first half of the Finnish words and to become familiarized with the fMRI tasks in a mock scanner. During session 3, participants will begin by learning the second half of the Finnish words, outside the MRI scanner. Once the learning tasks have been completed, participants will perform the 4AFC test while in the MR scanner. Once outside the scanner, participants will perform the Cued Recall task. Finally, in session 4, participants will be familiarized with the artificial language learning task in the mock scanner, and then proceed to perform the artificial language learning task in the MR scanner. In the grammar project (light blue arrows), participants will receive the test battery and a short Icelandic word training during session 1. In the following session, participants will undergo a short grammar training, with half of the learned words, to familiarize themselves with the Icelandic grammar rules before they will perform the grammar judgment task in the MRI scanner. After this session, participants will undergo a 5-day training, ~ 30 min each day, to learn the Icelandic grammar rules further as well as some additional Iceland words that will not be used in the grammar training. Finally, in the last session, participants will complete the second grammar judgment task on the grammar trained and untrained Icelandic words. Besides the task-based fMRI data, additional MR data will be collected during both MRI sessions

There are two subprojects, one on grammar learning and one on word learning. Each will randomly recruit 180 participants. Both projects will implement the same test battery and MRI sequences, but will differ on the fMRI paradigm and the corresponding training sessions (i.e., session 2 and home-training; see Fig. 1 and Table 1). Each fMRI paradigm is specifically developed to measure the neural correlates necessary for either L2 word learning (i.e., Finnish), L2 grammar learning (i.e., Icelandic), or L2 knowledge acquisition (i.e., artificial language task). All fMRI paradigms, as well as the rest of the protocol, are developed and tested with several pilot studies to ensure high-quality data can be collected for both young and adult participants.

**Table 1:** Study procedure chart for test battery and MRI protocol

Test battery	MRI protocol
**Cognitive tasks**	**1st MRI session**
*Matrix reasoning*	*MP2RAGE*
*Proficiency test L1 & L2*	Word learning fMRI task^a^
*Grammar test L1 & L2*	Grammar fMRI task: pre^b^
*Fluency test L1 & L2*	*Diffusion weighted imaging*
*ASRT task*	
*Word Pairs*	**2nd MRI session**
*Digit Span*	*MPRAGE*
*Non Word repetition task*	ArtLang fMRI task^a^
*StopIt Task*	Grammar fMRI task: post^b^
*Reading task*	*Resting-state fMRI*
**Questionnaires**	
*Socio-economic status*	
*Puberty development scale*	
*Handedness inventory*	
*Language experience*	

Study procedure chart showing research assessments for the word subproject (^a^), the grammar subproject (^b^), and overlapping—identical—assessments for both subprojects (italic). L1 = native language, L2 = second language, i.e., English

#### Cognitive tests

##### Language proficiency in Dutch and English

Dutch (L1) and English (L2) proficiency will be measured through a variety of standardized tests that measure vocabulary knowledge, grammatical ability, and word production in both languages. Vocabulary proficiency of each participant will be estimated using the standardized Peabody Picture Vocabulary Tests for the Dutch (PPVT-III-NL) and English (PPVT-4) language [127, 128]. Within the PPVT, each trial of the 204 or 228 trials in the Dutch and English versions respectively, presents visually four pictures and aurally one word. Participants have to select the picture that best matches the aurally presented word, which can be a verb, adjective, or noun. Grammar ability will be assessed using the—recently developed—Syntest and the test for reception of grammar (TROG-2) for the Dutch and English language respectively [129, 130]. In each grammar test, participants will match an aurally presented grammatical sentence with one of four presented pictures. The category fluency test and the initial letter fluency test will be employed to estimate the participants’ word production in Dutch and English [131]. For the category fluency test, participants need to generate exemplars from categories for vegetables and professions in Dutch, and animals and fruits in English, within 60 s per category. The initial letter fluency test requires participants to generate words from initial letters (i.e., Dutch: M and S; English: F and A) with a time limit of 60 s per letter.

##### Cognitive memory ability assessments

Several tests will be applied to assess individual variability in three main memory domains, that is, declarative, procedural, and short-term working memory. Declarative memory abilities of all participants will be measured through the word-pairs test from the Dutch version of the Wechsler scale of adult intelligence (WAIS; [129]). Procedural memory abilities will be estimated through an adapted version of the alternating serial reaction time (ASRT) task, where participants implicitly learn a repeated pattern of visual cue appearances that can occur in four different positions [132]. Short-term verbal memory will be assessed with the digit span test and the non-word repetition task (adapted from 134). The digit span test includes the backward, forward, and sorting task from the Wechsler scale of intelligence where adult participants will receive the WAIS-IV-NL and the younger participants until the age of 17 will receive t he WISC-V-NL version [133, 135].

##### General cognitive assessments

Various general cognitive assessments that measure non-verbal intelligence, cognitive control, and reading ability will also be collected. Non-verbal intelligence will be estimated using the matrices subtest from the Wechsler scale of intelligence for adults (WAIS-IV-NL) and children (WISC-V-NL), with the cut-off age of 17 years [133, 135]. To measure cognitive control, a stop-it task will be performed [136]. During this stop-it task, participants need to press either a left or right button when a square or circle is presented. However, this action has to be inhibited when the visual stimulus is accompanied by an auditory cue, that is, a loud beep. Reading ability will be checked using the Dutch standardized 3-min reading test, where participants have to read 120 verbs and nouns aloud within 3 min [137].

#### Questionnaires

Since several environmental and intrinsic factors could influence L2 learning, we will include a number of additional measures. Quantitative data about physical development, social-economic status, handedness, and language experience will be collected through various questionnaires. First, to assess physical pubertal development, all participants under 18 years will be asked to fill out a standardized pubertal development questionnaire, where their bodily maturation will be assessed through five questions with a 5-point Likert scale [138]. Secondly, participants or their caregivers (in case of underage participants) will be given a questionnaire about their income, education, and occupational status, to estimate their socio-economic status. Also, handedness will be measured through a questionnaire, namely through a Dutch version of the Edinburgh Handedness Inventory [139]. In addition to the previously mentioned Dutch and English proficiency tests, we will also ask the participants about their usage and experience with any foreign languages through an extensive language questionnaire.

#### Neuroimaging

All neuroimaging data will be acquired on a 3 T Siemens SKYRA MRI scanner using a 32-channel head coil at the Donders Institute for Brain, Cognition and Behaviour in Nijmegen. To minimize head movements, a small pillow will be placed in the head coil to stabilize the participant’s head and make them lie comfortably. Furthermore, a small skin-friendly tape will be placed on the forehead of each participant and the head coil base to provide them with head motion feedback, which is a child-friendly way to prevent head movement. Before each scan session, participants will be invited to lie in a mock scanner to acclimatize to the MRI scanner and the previously mentioned procedures.

##### Gray matter structural measurements

We will collect two whole-brain T1-weighted images during which participants are asked to lie as still as possible while watching short cartoons. One structural T1-weighted image will be acquired using a Magnetization Prepared Rapid Gradient Echo (MPRAGE) sequence. This sequence will allow for gray-matter density—that is, volume—measurements, but more importantly these images will serve as anatomical reference for co-registration and normalization of all neuroimaging data collected within the study. The parameters for this MPRAGE sequence are as follows: repetition time (TR) = 2000 ms; echo time (TE) = 2.01 ms; matrix size = 256 × 256; field-of-view (FOV) = 256 mm; flip angle = 8°; voxel size = 1 mm; slice thickness = 1 mm, 192 sagittal slices covering the entire brain. Parallel imaging (iPAT = 2) will be used to accelerate the acquisition, resulting in an acquisition time of 4 min and 40 s. The other high resolution T1-weighted image in this protocol is the MP2RAGE, a modified in-ho use M PRAGE sequence, which generates two different images at different inversion times [140]. This sequence is added to examine the properties of the gray matter beyond volume (i.e., surface and thickness) more precisely. The MP2RAGE sequence parameters are as follows: TR/TI1/TI2 = 5000/700/2500 ms; matrix size = 256 × 216; FOV = 256 mm; flip angle1 = 6°; flip angle2 = 5°; voxel size = 1 mm; slice thickness = 1 mm, 224 sagittal slices coveri ng th e entire brain. Parallel imaging (iPAT = 4.6) will be used to accelerate the acquisition, resulting in an acquisition time of 4 min. A corresponding low-resolution B1 fieldmap will be collected subsequently to correct for small inhomogeneities [141]. A total of 42 sagittal slices will be acquired, covering the entire brain in an acquisition time of 20 s. The B1 fieldmap sequence parameters are as follows: TR = 1000 ms; TE = 2.07 ms; matrix size = 256 × 216; F OV = 256 mm; voxel size = 1 mm; slice thickness = 1 mm.

##### White matter structural measurements

To examine white matter structures, we will acquire diffusion-weighted images. This acquisition will consist of a two-shell protocol with gradient directions that were uniformly distributed over the sphere: TR = 2930 ms; TE = 89.6 ms; multiband acceleration factor = 3; FOV = 212 × 212 × 138 mm, 2.0 mm^3^ isotropic voxels; 69 slices; 80 directions with b = 1000, 2000 s/mm^2^ (40 per b-value), and six b = 0 volumes, GRAPPA factor 2, phase partial Fourier Off; and a total acquisition duration 4:53 min. To allow for offline distortion correction of the diffusion-weighted images, 7 more b0 s/mm^2^ volumes will be acquired (duration 1:02 min) using exactly the same sequence parameters except for the inverted k-space read-out trajectory.

##### Resting-state functional MRI measurements

To obtain the resting-state data, a multiband accelerated T2*-weighted EPI sequence will be used with the following parameters: TR = 1000 ms; TE = 35.2 ms; multiband acceleration factor = 6; matrix size = 104 × 104; FOV = 213 mm; flip angle = 60°; voxel size = 2 × 2 mm; slice thickness = 2 mm, 66 axial slices covering the entire cerebrum. The duration of the scan is 8 min and 10 s, in which 480 volumes are obtained. Additionally, fieldmap data (one phase difference image and two magnitude images at two different echo times) will be acquired for distortion correction. Participants will be instructed to relax, lie as still as possible, and remain awake. During the scan, lights will be 50% dimmed and participants can either fixate on a white cross that is presented on a black screen in the scanner, or they can keep their eyes closed.

##### Task-based functional MRI measurements

This study protocol will implement three task-based fMRI paradigms with an identical multi-echo multiband T2*-weighted EPI sequence, only the duration differs depending on the length of each paradigm. This sequence enhances the exploration of deep gray matter structures and prefrontal and temporal brain regions and consist of the following parameters: TR = 1500 ms; TE1 = 12.4 ms, TE2 = 34.3 ms, TE3 = 56.2 ms; s lices = 51, interleaved slice order; slice thickness = 2.5 mm; multiband acceleration factor = 3; FOV = 210 × 210 × 128 mm; flip angle = 75°; voxel size = 2.5 × 2.5 × 2.5 mm. Slices will be angulated in an oblique axial manner to reach whole-brain coverage (except for a part of the cerebellum). Additionally, fieldmap data consisting of one phase difference image and two magnitude images at two different echo times will be acquired for distortion correction: TR = 576 ms; TE1 = 4.3 ms, TE2 = 6.79 ms; slices = 51, interleaved slice order; slice thickness = 2.5 mm; bandwidth = 804 Hz/Px; FOV = 210 × 210 × 128 mm; voxel size = 2.5 × 2.5 × 2.5 mm.

##### fMRI paradigms: word learning subproject

The neural correlates underlying word learning will be investigated using two fMRI tasks. Task 1—the word learning task—is a 4AFC task assessing the memory for Finnish words that participants learn during the course of the study. Task 2—the artificial language learning task—assesses knowledge accumulation and updating, two parameters that are estimated using a State Space model.

##### fMRI task 1: word learning paradigm

In this task, participants learn Finnish words. The number of words each participant will learn depends on their age to keep task difficulty relatively s imilar across the age groups. This approach was previously employed in a study comparing children and adults on their memory ability [142]. Fifty percent of the words that will be learned during these sessions are Finnish words for known objects such as belt, hammer, or tent. The remaining Finnish words are words for objects that are unknown to all participants. These unknown objects are pieces of ancient farming equipment [143]. Words will be taught on two separate days, during session 2 (remote) and during session 3 (recent; see Fig. 1).

**Fig. 2:**
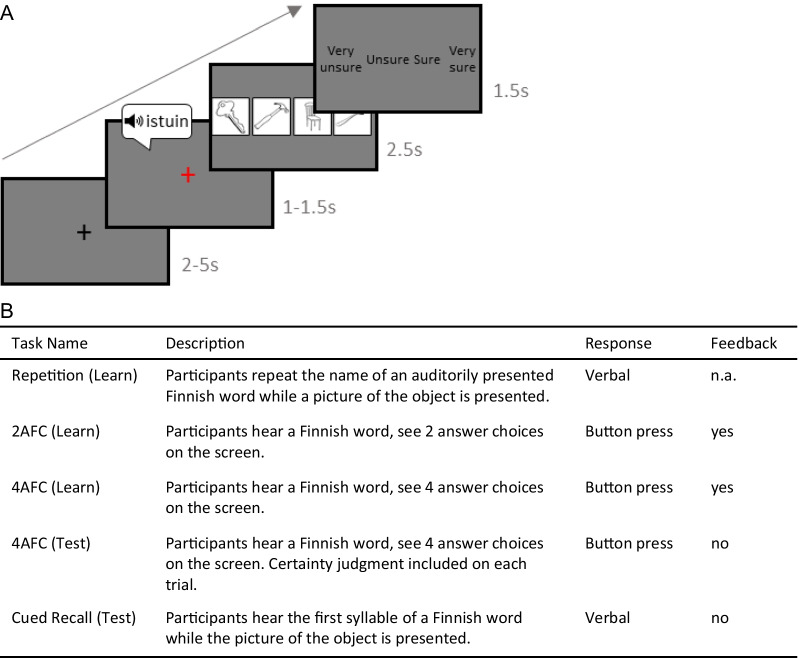
Word learning paradigm. **A** Example trial of Finnish word learning fMRI task. After the fixation cross turns red, the participants will hear a learned Finnish word through headphones. Next, participants will choose the correct of four pictures by button press. Finally, the participants will rate the certainty of their response. **B** Training and test tasks of the word learning fMRI-paradigm

Participants will learn the Finnish words through three learning tasks performed in both sessions 2 and 3, outside the scanner, see Fig. 1. Memory for Finnish words learned during sessions 2 and 3 will be assessed using the 4AFC-test in the fMRI task, which will be acquired after the learning tasks during session 3. Prior to this, the participants will have been familia rize d with the 4A FC-te st f MRI tas k during a dumm y scanner session. The task consists of up to 128 trials. Each trial of the 4AFC-test task will start with an auditory presentation of one Finnish word, and will be followed by 4 images appearing on the screen. Participants wi ll choose the matching object by means of button press. Finally, participants will indicate the certainty of their response, see Fig. 2. After the scan during session 3, participants will perform the Cued Recall task.

##### fMRI task 2: artificial language paradigm (ArtLang)

**Fig. 3:**
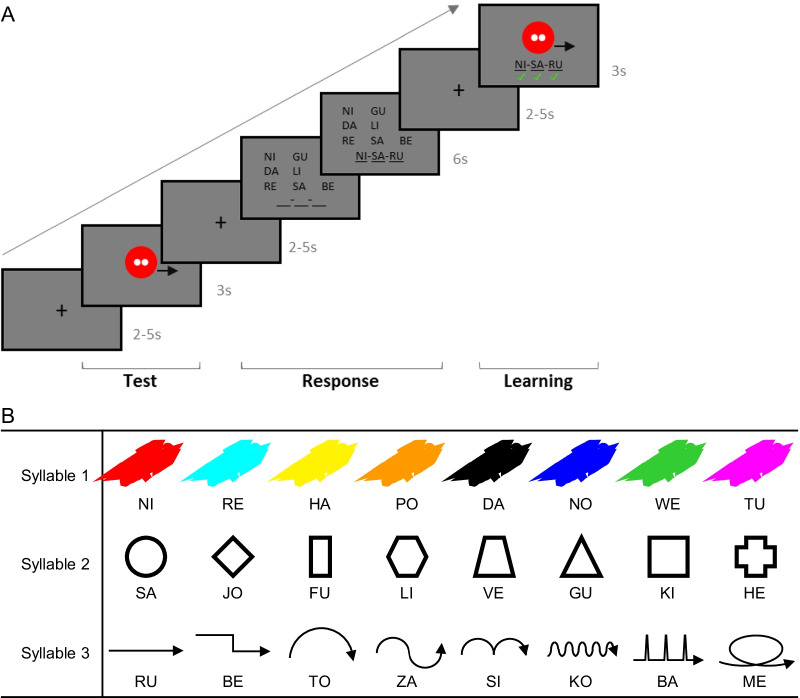
Artificial language learning paradigm. **A** Example trial of artificial language learning fMRI task (Level 3). Test: stimulus presented; Response: response made for each syllable; Learning: stimulus re-presented, with feedback provided under each syllable. **B** Overview of colors, shapes and movements used to create the aliens, as well as the syllables used to compose the names of the aliens. Syllable assignment as well as the combination of features was randomized

This artificial language learning task is an adapted version of the task that has previously been successfully implemented with adult participants [144, 145]. Participants will gradually learn names of aliens (that are composed of up to three syllables), which are based on three features of the aliens: color, shape, and movement, see Fig. 3B. The basic setup of all trials is the same, see Fig. 3A. Participants will see a colored, moving shape that is introduced as an alien. After this, they will see a matrix of possible answers and will be asked to indicate the correct syllable(s) using button press(es). Participants will then see the same colored, moving shape again and the alien’s correct name is presented.

The task consists of up to 3 levels and has 60 trials. During level 1, participants will learn the first syllable of the aliens’ names. When participants meet the learning criterion for level 1, i.e. two of the last three trials were answered correctly for three out of t he four colors presented in level 1, they will move to level 2. In level 2, participants will learn the second syllable. When participants meet the learning criterion for level 2, i.e. six of the last nine trials were answered correctly, they will move to level 3 (for more task details see Additional file 1). The adaptive nature of this task enables difficulty to be kept similar across participants. Participants will be explicitly instructed at the beginning of the task that the first syllable of the alien’s name refers to its color, the second to its shape, and the third to its movement—the instruction for the third syllable is only given when participants reach level 3.

##### fMRI paradigm: grammar learning subproject

**Fig. 4:**
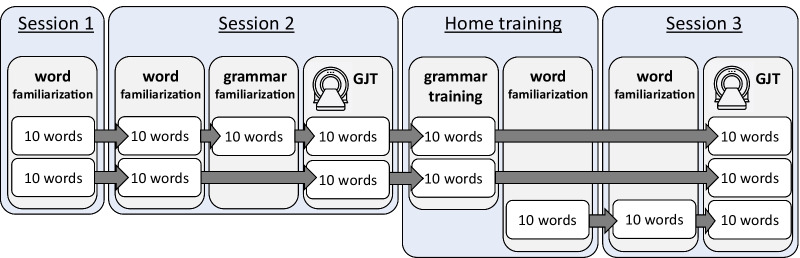
Schematic overview of Icelandic words within the familiarization, training sessions and grammar judgment tasks (GJTs). Before each GJT fMRI session, participants will be familiarized with all the words (either 20 or 30) that are included into the GJT, this familiarization is repeated a day later to allow for overnight consolidation. Participants will train the grammar rules on only a part of these words, i.e., 50% for the first GJT and 75% for the second GJT. Each GJT contains 192 trials, of which half contains words that were not trained during the grammar training

To assess the neural correlates of novel grammar learning, participants will perform a grammar judgment task (GJT) twice, once before and once after 5 days of grammar training at home. The Icelandic words used during grammar learning are cognates in Dutch (for the full words list  see Additional file 2); to enhance the readability of the Icelandic words diacritics were removed and non-Dutch letters, such as 'ð' and 'æ', were replaced with typical Dutch letters, such as 'd' and 'ae'. Participants will be familiarized with these Icelandic words through a word-picture memory game prior to the first and second GJT (see Fig. 4 and Additional file 4). Before the first grammar judgment task, participants will complete a short grammar familiarization training where they are implicitly familiarized with grammar rules of the Icelandic language: inflection of word phrases based on gender (masculine/feminine), number (singular/plural), and case (nominative/accusative; for an overview of all the rules see Additional file 3). This familiarization training consists of three blocks, each block consisted of 44 familiarization trials followed by 16 test trials. The familiarization trials contain Icelandic sentences and images, where the participant will have to correctly match either inflected words phrases with images or vice versa. The inflection rules will not be explained, but the correct answer will be shown after each trial, irrespectively of the given answer. For wrongly answered trials, the program additionally gives feedback that the given answer is incorrect. The test trials are Icelandic sentences that have to be judged on correctness, and are added to measure the participants’ improvement during the familiarization training. Since this study includes both adults and young children who differ in cognitive capacities, we have implemented three levels of the grammar difficulty to avoid ceiling or floor effects in learning performance across ages: In level 1, participants will learn the grammar rules for the Icelandic numerals and nouns, whereas in level 2 and level 3 participants will learn the adjectives for masculine nouns and all nouns, respectively (for more details see Additional file 3). During the familiarization training, participants will start in level 1 and, at the end of each block, move to the next level if they meet the learning criterion (i.e., ≥ 75% correct test trials) or otherwise continue the next block in the same level. At the end of the familiarization training, participants will stay in their reached level (i.e., 1, 2 or 3) for both fMRI tasks and the home training.

Between th e two MRI sessions a home training is planned: participants will play a grammar learning game for 30 min each on five consecutive days, to implicitly learn and train the Icelandic grammar rules for the previously learned 20 Icelandic words (for a detailed description see Additional file 4). Each day of the game consists of 3 blocks of 30 training items, identical to the familiarization training, and 16 test items. The test items are Icelandic sentences that have to be judged on correctness, just like the grammar judgment paradigm, and are added as a control measurement of the participants’ improvement during the home training.

##### fMRI task 3: grammar judgment paradigm

**Fig. 5:**
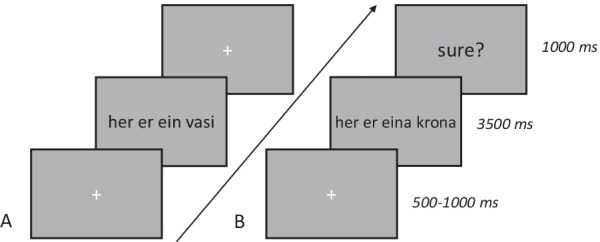
Grammar judgment tasks. Example trials of the grammar judgment task with Icelandic stimuli during the **A** pre-training fMRI session and **B** post-training fMRI session. For each fMRI task, each trail starts off with a fixation cross, the participants will read an Icelandic sentence and judge the correctness of the sentence. During the post-training fMRI task, the participants will additionally rate the certainty of their response

During the grammar judgment paradigm, participants will have to judge whether the presented Icelandic sentences are grammatically correct, indicating their answer by a left-hand button press. The task consists of 192 trials divided over a baseline and three grammar conditions. The first GJT includes 20 Icelandic words, of which 50% will have been used during the grammar familiarization training, for the second GJT all Icelandic words are included, of which 20 words will have been used during the grammar home training. The baseline (B) is a sensory-motor decision task, where the task is to indicate whether two Icelandic words are identical or different. The other three conditions consist of three types of Icelandic sentences that either follow (~ 67%) or violate (~ 33%) the learned rules . These sentences are either (C1) non-inflected, (C2) number inflected, or (C3) case inflected. Each trial consists of a white fixation cross, followed by an I celandic sentence presented on a light-gray screen for 3500 ms and ends with another white fixation cross for 1000 ms, see Fig. 5A). To maximize the power of the design, the intertrial interval and trial randomization is optimized using optseq2 [146]. After the 5-day training, participants will perform another GJT, which is identical to the first GJT except that the participants will indicate the certainty of their response at the end o f each trial, replacing the initial 1000 ms fixatio n cross (see Fig. 5B). For both sessions, reaction time and accuracy will be monitored throughout the experiment. Before each MRI session, all participants will be trained on a computer where they will receive task instructions and test material. Participants that are new to the MRI scanner will be trained using a dummy MRI scanner, where they can get used to the scanner sounds and practice with the MRI-compatible response buttons.

### Statistical analyses

To examine the neural correlates underlying age-related variability in new word and grammar learning, several analyses will be performed, which can be divided into two stages. The first stage is to estimate the cognitive and neural fingerprints that will be fed into the statistical analyses. For the neural fingerprint of word and grammar learning, the following functional and structural correlates will be estimated for all participants in each subproject: (a) network of functional regions (de)activated during the language fMRI paradigms, (b) functional connectivity nodes and maps, (c) white matter metrics (e.g., diffusion tensor and tractography measures), and (d) gray matter metrics (e.g., cortical thickness, surface area). To estimate the cognitive fingerprint that will serve as the predictor for individual differences in new word and grammar learning, the cognitive and questionnaire measurements will be inputted into principal component analyses to create combined factor scores, such as L1 factors (Dutch proficiency), L2 factors (English proficiency), and language aptitude. In the second stage, with these fingerprints, we will first explore potential age-related effects within each modality using regression analyses where age—as a continuous variable—will be the predictor for the earlier assessed functional and structural metrics. For this, both linear and non-linear effects will be examined by fitting linear and non-linear models using generalized linear models (GLMs) and generalized additive models (GAMs). Next, linked independent component analyses will be used to integrate all imaging modalities. This data-driven approach provides an automatic decomposition of the images into spatial components characterizing the intersubject brain structural variability, i.e., each spatial component represents a mode of variation of brain structure and function across all participants [147]. Post hoc multiple (linear/non-linear) regression analyses will investigate the relationship between the identified spatial components, age and cognitive factors. Lastly, structural equation modeling will be used to investigate which fingerprint features (such as age, cognitive factors, and functional and structural metrics within the identified spatial components) best predict new word or grammar learning success.

## Discussion

The present study will be one of the largest and most comprehensive studies investigating the developmental shift and individual differences in new language learning, covering preadolescence to adulthood in a cross-sectional manner. The two main objectives of the study are to examine the neurocognitive developmental trajectory in new language learning and to identify factors that are associated with individual variability in language learning success. Exploring L2 learning variability across adolescence and early adulthood will provide an important step toward understanding the underlying mechanisms. To fully understand the underlying mechanisms of L2 learning, we combine the fields of memory, language, and neurodevelopment. Therefore, our study protocol involves multiple study visits containing comprehensive neurocognitive assessments, questionnaires, and extensive magnetic resonance imaging (MRI) protocols. The combination of several MRI modalities will allow us to meticulously explore the developmental trajectory in L2 learning. With this comprehensive data collection we will create behavioral and neural fingerprints that capture age-based variability and individual variability in new language learning.

## Strengths and limitations

The study design has a number of strengths. The large sample size of 360 participants, evenly spread over a broad age range (i.e., preadolescence until adulthood) is one of the most important strengths. This allows for more powerful analyses, including flexible correlational analyses, which surpass standard group comparisons. Thus far, studies have typically used small groups or only adults to make inferences about age-related variability in L2 learning. Furthermore, our strict inclusion criteria will create a homogenous sample in relation to native language (i.e., Dutch), bilingualism, and exposure to the new languages learned in the fMRI paradigms. Another strength of this study is that age-related and individual variability are measured on both a cognitive and neural level, which will contribute to an improved understanding of the factors influencing L2 learning success. For example, we measure language aptitude, memory skills, and higher executive functioning. Moreover, this will be the first study that examines the underlying mechanisms of L2 learning using a combination of multiple MRI techniques and ne urocognitive assessments within the same sample.

This study employs self-selecting sampling; participants will be voluntarily recruited from the general population t hrough promotion material at schools, libraries, sport clubs, and higher education recruitment systems. Our recruitment strategy could, therefore, lead to a biased sample of high-functioning, high-educated, and motivated participants with a high socioeconomic status (SES). This may limit the generalization of our results to the general population. Interestingly, the literature indicates that SES, motivation, and education level are factors that could influence L2 learning. For this r eason, we have included meas ures for SES, motivation, and education level in our study protocol. Although the chosen cross-sectional study design allows us to investigate age-related differences in a large sample within a short timeframe, it does have one important shortcoming. That is, the direct cause-and-effect of development on L2 learning success is difficult to pin down due to inter-individual variability. To compensate for this, we plan to collect a large sample. Additionally, we will measure and p otentially correct for developmental and environmental factors to reduce inter-individual variability when investigating age-related effects. Another limitation is the multilingual population of the Netherlands. In particular, individuals with a high SES learn multiple languages in high school and use English on a daily basis in higher education. Luckily, however, children in the Netherlands are already exposed to English and other languages at a young age and thus even our youngest participants ar e not monoling ual. But the children still have less L2 exposure than older individuals. This could cause differences in language knowledge between the preadolescents and young adults. To deal with this limitation, our protocol contains an extensive language questionnaire that measures English (and other) language knowledge and exposure, which will be factored into our analyses.

Our comprehensive approach of combining cognitive and neuroimaging data will contribute to the understanding of the mechanisms underlying the developmental shift previously observed in the L2 acquisition literature. The results of this study provide a unique opportunity to understand how the development of brain structure and function influence L2 learning. Furthermore, analyses focusing on cognitive factors within the memory and language domain will broaden our understanding about the individual differences in L2 learning success, irrespective of age. Our unique dataset will be made available for data sharing with other researchers in the fields of language, memory, and development.

## Supplementary Information

**Additional file 1.** Overview of adaptive set-up of the artificial language learning task.

**Additional file 2.** List of Icelandic nouns.

**Additional file 3.** Icelandic grammar rules.

**Additional file 4.** Example of the Icelandic word familiarization and grammar training tasks.

## Data Availability

Not applicable.

## Abbreviations

AF: Arcuate fasciculus
AoA: Age of acquisition
ArtLang: Artificial language paradigm
ASRT: Alternating serial reaction time
fMRI: Functional magnetic resonance imaging
GJT: Grammar judgment task
IFG: Inferior frontal gyrus
IFOF: Inferior fronto-occipital fasciculus
IPL: Inferior parietal lobe
L1: First language
L2: Second language
MFG: Middle frontal gyrus
MPRAGE: Magnetization Prepared Rapid Gradient Echo
MRI: Magnetic resonance imaging
MTG: Middle temporal gyrus
rsfMRI: Resting-state fMRI
SES: Socioeconomic status
SLF: Superior longitudinal fasciculus
STG: Superior temporal gyrus
TE: Echo time
TR: Repetition time

## Footnotes

1 We refer to new language learning instead of L2 learning to describe the language learning (i.e., tasks and trainings) within our project. With L2, we are referring to the general literature about second language acquisition.
