# Supplementary material for: Study protocol: a comprehensive multi-method neuroimaging approach to disentangle developmental effects and individual differences in second language learning
Source: BMC Psychol. 2022 Jul 8;10:169. doi: 10.1186/s40359-022-00873-x (PMC9270835; doi:10.1186/s40359-022-00873-x)
Supplement: Supplementary file 1 — Additional file 1. Overview of adaptive set-up of the artificial language learning task. [file 40359_2022_873_MOESM1_ESM.docx]

**Additional file 1.** **Overview of adaptive set-up of the artificial language learning task.**

**
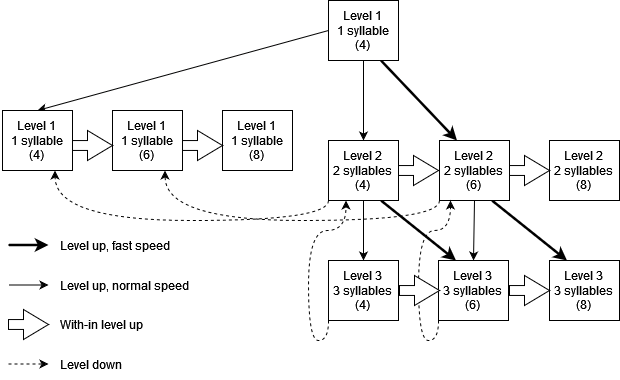
**

***Figure S1. Overview of adaptive set-up of the artificial language learning task.*** *All participants start at the top of the diagram in Level 1 with 4 colors (denoted by the number in parentheses). Depending on the speed of learning between the pairing of syllable and color, participants either progress to Level 2 with 6 colors and 6 shapes (level up, fast speed), Level 2 with 4 colors and 4 shapes (level up, normal speed), or if they do not reach the learning criterion of Level 1 before trial 30, they remain in Level 1 for the remainder of the task. The progression from Level 2 to Level 3 is similar to that from Level 1 to Level 2. If a participant performs poorly in a given level, i.e. no correct syllables during 4 consecutive trials, they move to the level below with the same number of features (Level down); e.g. a participant that is in Level 2 (6) will move down to Level 1 (6)****.***
