# Supplementary material for: Study protocol: a comprehensive multi-method neuroimaging approach to disentangle developmental effects and individual differences in second language learning
Source: BMC Psychol. 2022 Jul 8;10:169. doi: 10.1186/s40359-022-00873-x (PMC9270835; doi:10.1186/s40359-022-00873-x)
Supplement: Supplementary file 2 — Additional file 2. List of Icelandic nouns. [file 40359_2022_873_MOESM2_ESM.docx]

**Additional file 2. List of Icelandic nouns.**

**Table S1. List of Icelandic nouns.**

| Masculine | | | Feminine | | |
| --- | --- | --- | --- | --- | --- |
| # | Icelandic | *English* | # | Icelandic | *English* |
| 1 | **api** | *ape* | 11 | **blussa** | *blouse* |
| 2 | **jakki** | *jacket* | 12 | **flauta** | *flute* |
| 3 | **lampi** | *lamp* | 13 | **krona** | *crown* |
| 4 | **penni** | *pen* | 14 | **panna** | *pan* |
| 5 | **vasi** | *vase* | 15 | **sapa** | *soap* |
| 6 | **sofi** | *sofa* | 16 | **terta** | *cake* |
| 7 | **bolti** | *ball* | 17 | **taska** | *bag* |
| 8 | **dreki** | *dragon* | 18 | **flaska** | *bottle* |
| 9 | **jeppi** | *jeep* | 19 | **pumpa** | *pump* |
| 10 | **mani** | *moon* | 20 | **klukka** | *clock* |
|  |  |  |  |  |  |
| 21 | **bursti** | *brush* | 26 | **kirkja** | *church* |
| 22 | **falki** | *falcon* | 27 | **pera** | *pear* |
| 23 | **pakki** | *package* | 28 | **tromma** | *drum* |
| 24 | **safi** | *juice* | 29 | **stjarna** | *star* |
| 25 | **hani** | *rooster* | 30 | **kista** | *trunk* |

*The participants will learn in 30 Icelandic words (50% feminine) in total. They will learn the first 20 words (#1 to #20) during the initial training session, these words are included in the first grammar judgment task (GJT). At the end of the home training, they learn 10 additional words. During the second GJT, all words (#1 to #30) are included. Participants will only train with part of the words during the grammar training, that is 50% for the first GJT and then 75% of the words in the second GJT.*
