# Supplementary material for: Study protocol: a comprehensive multi-method neuroimaging approach to disentangle developmental effects and individual differences in second language learning
Source: BMC Psychol. 2022 Jul 8;10:169. doi: 10.1186/s40359-022-00873-x (PMC9270835; doi:10.1186/s40359-022-00873-x)
Supplement: Supplementary file 3 — Additional file 3. Icelandic grammar rules. [file 40359_2022_873_MOESM3_ESM.docx]

**Additional file 3. Icelandic grammar rules.**

**Table S2. Icelandic grammar rules.**

| rules | gender | numeral | adjective | noun | example sentence | English translation |
| --- | --- | --- | --- | --- | --- | --- |
| **nominative singular** | M | ein | -ur | -i | *her er* ein gulur penni | *here is a yellow pen* |
|  | F | eina | - | -a | *her er* eina gul krona | *here is a yellow crown* |
| **nominative plural** | M | tveir | -ir | -ar | *her eru* tveir gulir pennar | *here are two yellow pens* |
|  | F | tvaer | -ar | -ur | *her eru* tvaer gular kronur | *here are two yellow crowns* |
| **accusative singular** | M | einn | -an | -a | *her serdu* einn gulan penna | *here you see a yellow pen* |
|  | F | eina | -a | -u | *her serdu* eina gula kronu | *here you see a yellow crown* |

*Examples of the Iceland grammar rules used for the inflection of the 30 Icelandic nouns and 4 Icelandic adjectives (i.e., yellow, green, white, black). In level 1 participants will learn the grammar rules for the Icelandic numeral and nouns, whereas in level 2 adjectives for masculine words and in level 3 adjectives for all words are added.*
