# Supplementary material for: Study protocol: a comprehensive multi-method neuroimaging approach to disentangle developmental effects and individual differences in second language learning
Source: BMC Psychol. 2022 Jul 8;10:169. doi: 10.1186/s40359-022-00873-x (PMC9270835; doi:10.1186/s40359-022-00873-x)
Supplement: Supplementary file 4 — Additional file 4. Example of the Icelandic word familiarization and grammar training tasks. [file 40359_2022_873_MOESM4_ESM.docx]

**Additional file 4. Example of the Icelandic word familiarization and grammar training tasks.**


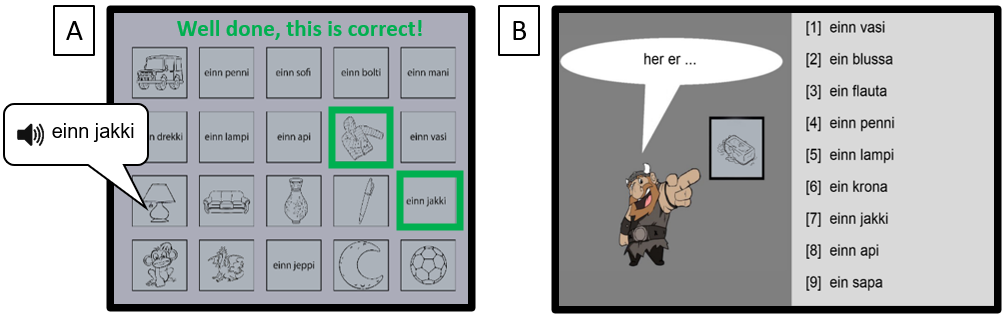


**Figure S2. Example of the Icelandic word familiarization (A) and grammar training (B) tasks.**

The word familiarization task is a visual memory game that consist of multiple squares containing Icelandic words or images. The goal is to pair the correct images with the Icelandic words. When a word-picture pair is assembled the Icelandic word is presented aurally. If the Icelandic word is paired correctly with the picture, both squares are outlined with a green color and ‘Well done, this is correct’ is presented. When an incorrect pair is assembled, then both squares are outlined with a yellow color and ‘Unfortunately, this is not correct’ will be presented. This task contained multiple memory boards on which all Icelandic words are repeated three times. To make sure all words were learned properly, each memory board was repeated with shuffled cards until no mistakes are made in that specific board.

The grammar training task consisted of three blocks. Each block starts with 30 training trials, where participants have to finish a sentence with the correct Icelandic word phrase by selecting one of the nine answers. Alternating trials were added where participants received the full Icelandic sentence and had to select the correct image. This was done to make the training more entertaining and keep motivation high, especially for the younger participants. Each block ends with 16 test trials, where participants have to judge whether Icelandic sentences were correctly written. These test trials are added as a control measurement of the participants’ improvement during the training.
